# Potency of *Lacticaseibacillus paracasei* as an alternative to antibiotic growth promoter in broiler chicken challenged with avian pathogenic *Escherichia coli*

**DOI:** 10.14202/vetworld.2025.1180-1189

**Published:** 2025-05-17

**Authors:** Widya Paramita Lokapirnasari, Lilik Maslachah, Amung Logam Saputro, Efi Rokana, Andreas Berny Yulianto, Zulfi Nur Amrina Rosyada, Muhammad Aviv Firdaus, Himatul Ilma Silfia, Ertika Fitri Lisnanti, Zein Ahmad Baihaqi, Tabita Dameria Marbun, Aswin Rafif Khairullah, Muhammad Shakeel

**Affiliations:** 1 Department of Animal Husbandry, Faculty of Veterinary Medicine, Universitas Airlangga, Jl. Dr. Ir. H. Soekarno, Kampus C Mulyorejo, Surabaya 60115, East Java, Indonesia; 2 Department of Basic Veterinary Medicine, Faculty of Veterinary Medicine, Universitas Airlangga, Jl. Dr. Ir. H. Soekarno, Kampus C Mulyorejo, Surabaya 60115, East Java, Indonesia; 3 Veterinary Medicine Study Program, Faculty of Health, Medicine, and Life Sciences, Universitas Airlangga, Jl. Wijaya Kusuma No.113, Mojopanggung, Giri, Banyuwangi 68425, East Java, Indonesia; 4 Division of Animal Husbandry, Faculty of Agriculture, Universitas Islam Kadiri, Jl. Sersan Suharmaji No.38, Manisrenggo, Kota, Kediri 64128, East Java, Indonesia; 5 Faculty of Veterinary Medicine, Universitas Wijaya Kusuma Surabaya, Jl. Dukuh Kupang XXV No.54, Dukuh Kupang, Dukuh Pakis, Surabaya 60225, East Java, Indonesia; 6 Master Program of Veterinary Agribusiness, Faculty of Veterinary Medicine, Universitas Airlangga, Jl. Dr. Ir. H. Soekarno, Kampus C Mulyorejo, Surabaya 60115, East Java, Indonesia; 7 Research Center for Animal Husbandry, National Research and Innovation Agency (BRIN), Jl. Raya Bogor, Km. 46 Cibinong, Bogor 16911, West Java, Indonesia; 8 Animal Nutrition Laboratory, Kyungpook National University, 80 Daehak-ro, Buk-gu, Daegu, Sangju 37224, Korea; 9 Research Center for Veterinary Science, National Research and Innovation Agency (BRIN), Jl. Raya Bogor, Km. 46 Cibinong, Bogor 16911, West Java, Indonesia; 10 Faculty of Veterinary and Animal Sciences, Pir Mehr Ali Shah, Arid Agriculture University, Shamsabad, Muree Road, Punjab 46000, Pakistan

**Keywords:** antibiotic alternatives, antioxidant status, broiler chicken, Escherichia coli, growth performance, lipid metabolism, probiotics

## Abstract

**Background and Aim:**

The emergence of antibiotic-resistant bacteria due to the widespread use of antibiotic growth promoters (AGPs) necessitates the exploration of sustainable alternatives in poultry production. This study evaluated the efficacy of *Lacticaseibacillus paracasei* as a probiotic alternative to AGPs in broilers challenged with avian pathogenic *Escherichia coli* (APEC), with a focus on growth performance, antioxidant status, lipid metabolism, and hematological profiles.

**Materials and Methods:**

A total of 80 broiler chickens were randomly allocated into four groups: T0 (uninfected control), T1 (APEC-infected control), T2 (APEC + AGP, 0.1% zinc bacitracin), and T3 (APEC + probiotic, 0.5% *L. paracasei*). Treatments were administered from day 15 to 35 post-hatch, following a 2-week adaptation period. Performance indicators (feed conversion ratio [FCR], body weight gain [BWG], and feed efficiency [FE]), antioxidant parameters (superoxide dismutase [SOD] and malondialdehyde [MDA]), lipid profiles (cholesterol, HDL, and LDL), and hematological variables were assessed. Data were analyzed using analysis of variance with significance set at p < 0.05.

**Results:**

Probiotic supplementation significantly improved FCR, BWG, and FE (p < 0.05), with the T3 group achieving the most favorable outcomes. SOD activity was markedly higher, and MDA levels were reduced in probiotic-treated birds compared with the AGP and infected groups. Moreover, *L. paracasei* administration resulted in significant reductions in total cholesterol and LDL levels while maintaining moderate HDL concentrations. Hemoglobin and thrombocyte levels were modestly influenced, though overall hematological profiles remained stable across groups.

**Conclusion:**

*L. paracasei* supplementation significantly enhanced growth performance, antioxidant defense, and lipid metabolism in broilers challenged with APEC, supporting its application as a viable alternative to AGPs. These findings contribute to sustainable poultry production practices and public health safety by mitigating the reliance on antibiotics.

## INTRODUCTION

Colibacillosis, a poultry disease caused by avian pathogenic *Escherichia coli* (APEC), continues to represent a significant challenge in both Indonesia and the global poultry industry. The transmission of *E. coli* through contaminated animal products and fecal matter constitutes a substantial public health risk, leading to considerable economic losses attributed to reduced hatchability, diminished production yields, heightened morbidity and mortality rates, and increased veterinary and medical costs [1]. In alignment with Law No. 18 of 2009 in conjunction with Law No. 41 of 2014 concerning animal husbandry and health, particularly Article 22 paragraph 4C, which prohibits the use of feed additives containing specific hormones and/or antibiotics, there is an opportunity to develop effective alternatives to antibiotic growth promoters (AGPs) to enhance production while ensuring food safety for human consumption. To achieve these objectives, innovation in the development of feed additives substituting AGPs is urgently required [2].

AGPs have been widely utilized in poultry farming to enhance feed efficiency (FE) and accelerate growth rates, primarily through modulation of intestinal health and reduction of disease susceptibility [3]. Owing to these benefits, AGPs have served as indispensable components of intensive poultry production systems for several decades [3, 4]. However, the rising prevalence of antibiotic-resistant bacteria and the detection of antibiotic residues in meat products have increasingly raised public health and food safety concerns [5, 6]. As a result, many countries have adopted stringent regulations or have enacted outright bans on the use of AGPs in animal agriculture, thereby driving a global transition toward alternative strategies aimed at maintaining livestock productivity [7].

Among the alternatives to AGPs, probiotics, prebiotics, herbs, and plant extracts have been expl-ored, with probiotics emerging as the most widely accepted due to their demonstrated safety and effic-acy [8]. In poultry production, the administration of live microorganisms at adequate levels fosters the development of healthy intestinal microbiota, enhances disease resistance, reduces pathogenic colonization, improves nutrient assimilation, and strengthens host immunity [7, 9]. Consequently, probiotics have become integral to modern poultry production systems, offering the dual advantages of improving productivity while meeting consumer demands for antibiotic-free, safer meat products [10, 11].

Probiotics, particularly lactic acid bacteria (LAB), have gained prominence as potential replacements for AGPs due to their ability to promote gut health, enhance immune function, and improve overall production performance in poultry. LAB encomp-asses several genera, including *Lactobacillus*, *Lactococcus*, *Streptococcus*, *Carnobacterium*, and *Enterococcus* [12–15]. *Lacticaseibacillus paracasei* (*L. paracasei*), previously classified as *Lactobacillus paracasei*, belonging to the *L. casei* group, has been extensively studied for its probiotic properties, including its ability to colonize the gastrointestinal tract and its resilience under acidic conditions. In add-ition, *L. paracasei* has demonstrated capabilities in modulating immune responses, enhancing digestion, reducing inflammatory processes [16, 17], alleviating metabolic disorders [18], and lowering cholesterol and fasting blood sugar levels in BALB/c mice models [19]. Probiotic strains such as *L. paracasei* are commonly found in the gut flora of healthy individuals and tradi-tionally fermented foods, including cheese and yogurt. Moreover, dietary supplementation with LABs such as *L. paracasei* and *L. plantarum* has been shown to enhance poultry production performance and mitigate pathogenic bacterial loads in both healthy and infected bird populations [5, 20].

Despite substantial evidence supporting the efficacy of LAB, including *L. paracasei*, in enhancing gut health, modulating immunity, and improving overall production performance in poultry, specific studies evaluating the role of *L. paracasei* under conditions of APEC infection remain limited. Most existing research has focused on general probiotic benefits in hea-lthy broilers, with comparatively fewer investigations addressing their protective effects against bacterial challenges that mimic field conditions. Furthermore, while probiotics have demonstrated potential in modulating lipid profiles and antioxidant capacities, comprehensive assessments integrating growth perfo-rmance, antioxidant status, lipid metabolism, and hematological parameters in broilers challenged with APEC are scarce. This paucity of integrated studies limits the ability to position *L. paracasei* as a viable alternative to AGPs in high-risk infection scenarios within intensive poultry production systems.

Therefore, the present study aimed to evaluate the potential of *L. paracasei* supplementation as an alternative to AGPs in broilers experimentally challenged with APEC. Specifically, the study investigated the effects of *L. paracasei* on growth performance metrics (feed conversion ratio [FCR], body weight gain [BWG], FE), antioxidant defense mechanisms (superoxide dismutase [SOD] and malondialdehyde [MDA] levels), lipid metabolism (cholesterol, HDL, and LDL concentrations), and hematological profiles. By elucidating the multifa-ceted benefits of *L. paracasei* under infectious stress conditions, this research contributes to the development of sustainable strategies for poultry health management, addresses the global imperative to reduce antibiotic use in animal agriculture, and enhances food safety and public health outcomes.

## MATERIALS AND METHODS

### Ethical approval

Ethical approval for this study was obtained from the Animal Ethics Commission, Faculty of Veterinary Medicine, Universitas Airlangga (approval number 1.KEH.017.01.2024).

### Study period and location

The study was conducted at the Faculty of Veterinary Medicine, Universitas Airlangga, between June and October 2024. The experimental setup utilized APEC at a concentration of 1.5 × 10^8^ CFU/mL and *L. paracasei* at 1.2 × 10^9^ CFU/mL. Broilers were housed in battery cages measuring 35 cm × 20 cm × 45 cm and were provided with commercial feed (CP-511). A growth promoter (zinc bacitracin) was incorporated into the feed, and digital scales were used to measure both bird body weight and feed intake.

### Methods

A true experimental design with a completely randomized layout was employed to evaluate the effects of *L. paracasei* supplementation through drinking water and zinc bacitracin as an AGP incorporated into the feed. A total of 80 broilers were randomly allocated to four experimental groups, each consisting of 10 replicates, with two birds per replicate. The experimental groups were designed as follows:

- T0 (Negative control): Broilers without APEC infection.
- T1 (Positive control): Broilers infected with APEC.
- T2: Broilers infected with APEC and supplemented with AGP (0.1% zinc bacitracin).
- T3: Broilers infected with APEC and supplemented with probiotics (0.5% *L. paracasei*).

An adaptation period of 2 weeks (days 1–14) was followed by a 5-week feeding period, starting from day-old chick arrival and continuing until harvest at 35 days of age. Probiotic and AGP treatments were administered from day 15 to day 35. Broilers were fed twice daily at 08:00 AM and 03:00 PM using commercial feed (CP-511). Drinking water was made available *ad libitum*, with *L. paracasei* (0.5%) delivered through the drinking water and zinc bacitracin (0.1%) incorporated into the feed during the treatment period.

### Procedures for *E. coli*, AGP, and probiotic administration

At 21 days of age, broilers were orally challenged with APEC at a concentration of 10^8^ CFU/mL. The probiotic *L. paracasei* (formerly *L. paracasei*) was admin-istered at an optimal concentration of 0.5% through drinking water. Probiotics were prepared by accurately measuring the required quantities and thoroughly mixing them into the drinking water. Growth promoters (AGPs), specifically zinc bacitracin, were incorporated into the feed at a dose of 1 kg/ton, following the manufacturer’s recommendations, and were provided during regular feeding.

### Data collection

Data collection was performed following the standard methods of the Association of Official Analytical Chemists (AOAC). Growth performance parameters, including body weight, FCR, and FE, were recorded on a weekly basis. The FCR was calculated by dividing total feed intake (g) by BWG (g), with lower FCR values indicating better feed utilization. FE was determined as the ratio of BWG to total feed intake and expressed as a percentage, with higher FE values indicating improved FE.

Blood samples were collected on day 35 before sacrifice. The slaughter procedure was conducted thro-ugh exsanguination through the jugular vein, brachial vein (cutaneous ulnar vein), and ischiadic vein (sciatic vein), following standard protocols, with all 80 birds included in the analysis.

Cholesterol, high-density lipoprotein (HDL), and low-density lipoprotein (LDL) levels were assessed at the conclusion of the treatment period using a spectrophotometer, following the manufacturer’s protocol for the EnzyChrom™ AF HDL and LDL/VLDL Assay Kit (E2HL-100). In addition, blood serum samples were analyzed to determine antioxidant status. Filtrate extraction was performed for each treatment, with two meat samples and one blood serum sample collected per replicate [21, 22].

### Statistical analysis

All data collected were subjected to statistical analysis using a completely randomized design. The data were first evaluated for normality and homogeneity of variance (analysis of variance [ANOVA]). Statistical analyses were performed using the Statistical Product for Service Solution software, version 27 (IBM Corp., NY, USA). Differences among mean values were assessed using one-way ANOVA. Further comparisons among group means were conducted using Duncan’s multiple range test, with statistical significance considered at p < 0.05.

## RESULTS

### FCR, BWG, and FE

This study also evaluated the impact of *L. paracasei* supplementation on FCR, weight gain, and FE in broilers infected with *Escherichia coli*. Table 1 summarizes the results, indicating significant differences among the treatment groups in terms of FCR, weight gain, and FE. The T3 group, which received 0.5% *L. paracasei* supplementation in *E. coli*-infected broilers, exhibited the most favorable FCR at 1.43, significantly surpassing the other groups (p < 0.05). Probiotic supplementation enhances feed conversion in broilers during infection conditions. The T0 group, serving as the negative control for *E. coli* infection or feed additives, exhibited a comparable FCR of 1.53, akin to that of T3, although lacking the advantages conferred by probiotic supplementation. The T1 group, which was infected with *E. coli* but did not receive feed additives, exhibited an FCR of 1.57. Conversely, the T2 group, also infected with *E. coli* but supplemented with AGP (zinc bacitracin), recorded the highest FCR of 1.60. Nonetheless, no significant differences were detected among the T0, T1, and T2 groups (p > 0.05), indicating that the lack of probiotics and the application of AGP did not meaningfully affect FCR during *E. coli* infection.

**Table 1:** FCR, BWG, and FE.

Treatment	FCR	Weight gain (g/head)	FE (%)
T0	1.53^ab^ ± 0.06	458.80^b^ ± 27.87	65.42^a^ ± 2.49
T1	1.57^b^ ± 0.07	418.00^a^ ± 23.10	63.91^a^ ± 2.82
T2	1.60^b^ ± 0.08	432.40^ab^ ± 16.31	62.70^a^ ± 3.40
T3	1.43^a^ ± 0.06	520.20^c^ ± 39.21	69.82^b^ ± 2.75

^a,b,c^Different superscripts within a column indicate statistically significant differences between treatments (p < 0.05). FCR=Feed conversion ratio, BWG=Body weight gain, FE=Feed efficiency

Broilers in the T3 group exhibited the highest weight gain (520.20 g), demonstrating a statistically significant improvement over all other treatment groups (p < 0.05). These results suggest that *L. paracasei* supplementation significantly enhances the growth performance of broilers exposed to *E. coli*. Although the T0 group (non-infected, no feed additives) achieved a weight gain of 458.80 g, this was notably lower than the 520.20 g recorded in the T3 group, highlighting the probiotic’s efficacy in promoting superior growth outcomes. The T1 and T2 groups, infected with *E. coli* and receiving either no additives or AGP supplementation, exhibited the lowest weight gains of 418.00 and 432.40 g, respectively; however, the difference between these groups was not statistically significant. This finding indicates that probiotics in the T3 group were more effective for weight gain, whereas the effects of AGP in the T2 group were comparatively less effective.

The T3 group showed the highest FE of 69.82%, significantly surpassing the other groups (p < 0.05). The findings indicate that *L. paracasei* supplementation enhances feed utilization in broilers, particularly during *E. coli* infection. The FE of the T0 group was 65.42%, comparable to that of the T3 group, suggesting that the absence of *E. coli* infection may have facilitated improved feed utilization, although without the advantages of probiotics. In contrast, the T1 and T2 groups, both infected with *E. coli*, demonstrated reduced FE values of 63.91% and 62.70%, respectively, with no significant differences noted between the groups (p > 0.05). This suggests that *E. coli* infection has a negative impact on FCR and FE. The administration of probiotics (T3) positively influenced FCR, as evidenced by its lower value, and improved FE by increasing the ratio of BWG to total feed intake. In contrast, AGP supplementation (T2) did not significantly enhance FCR or FE.

### SOD and MDA Levels

The levels of SOD and MDA are presented in Table 2, which presents the effects of various treatments. SOD is an essential antioxidant enzyme that reduces oxidative stress, and its expression differed significantly across treatment groups. The T3 group exhibited the highest SOD activity (40.25 U/mL, p < 0.05), indicating that probiotic supplementation significantly enhanced antioxidant defense mechanisms. In contrast, the T1 group (*E. coli* infection only with no feed additives) exhibited the lowest SOD (6.41 U/mL). The T2 group (AGP only) showed higher SOD activities (14.78 U/mL) compared to T1 (6.41 U/mL), respectively, indicating comparable antioxidant responses between the probiotics and AGPs when administered individually. However, SOD activity in T2 remained lower than in T3 (40.25 U/mL), which received probiotic supplementation. The T0 group (negative control) exhibited moderate SOD activity (19.61 U/mL), exceeding that of T1, which may indicate a natural response to oxidative stress in the absence of intervention.

**Table 2:** SOD and MDA levels.

Variable	T0	T1	T2	T3
SOD (U/mL)	19.61^b^ ± 8.31	6.41^a^ ± 1.50	14.78^b^ ± 1.09	40.25^c^ ± 1.61
MDA (nmol/mL)	2.02^a^ ± 0.05	6.41^c^ ± 1.22	2.27^a^ ± 0.27	4.30^b^ ± 0.94

^a,b,c^Different superscript letters within a column indicate statistically significant differences between treatment groups (p < 0.05). SOD: Superoxide dismutase, MDA: Malondialdehyde

MDA is a biomarker of lipid peroxidation and oxidative damage, exhibiting significant variations among the treatments. The T1 group (*E. coli* infection with no feed additives) had the highest MDA level (6.41 nmol/mL; p < 0.05). In contrast, the T2 group (AGP) exhibited significantly reduced MDA levels (2.27 nmol/mL), which was comparable to the T0 group (2.02 nmol/mL). The T3 group (probiotic) showed intermediate MDA levels (4.30 nmol/mL), suggesting partial protection against oxidative damage when prob-iotics were administered.

The markedly lower MDA levels in the T2 group (AGP) highlight the ability of AGP to effectively mitigate oxidative damage, which is comparable to natural conditions (T0). The comparable SOD activity observed between the T2 and T1 groups indicates that AGP has an anti-oxidative capacity similar to that of negative controls.

### Cholesterol, HDL, and LDL levels in broiler

The T3 group (probiotic) showed the lowest cholesterol levels (50.26 mg/100g), which were significantly lower than those in all other groups (p < 0.05). The application of probiotics significantly reduced cholesterol levels in broiler meat. The T2 group (AGP) exhibited the highest cholesterol levels (139.55 mg/100 g), followed by the T1 group (102.66 mg/100 g) and the T0 group (76.86 mg/100 g). HDL levels were highest in the T0 group (61.48 mg/100 g), significantly exceeding those of all other groups. The T2 group (AGP) had the lowest HDL levels (14.90 mg/100 g), whereas the T1 (26.39 mg/100g) and T3 (32.80 mg/100 g) treatment groups had intermediate HDL levels. The T2 group (AGP) had the highest LDL-C levels (82.94 mg/100g), whereas the T0 group (negative control) had the lowest LDL-C levels (57.18 mg/100g). The T1 (74.82 mg/100g) and T3 (66.00 mg/100g) treatment groups exhibited intermediate LDL-C levels. The findings indicate that probiotic supplementation (T3) significantly reduced cholesterol (50.26 mg/100g) and LDL levels (66.00 mg/100g) compared with the AGP-treated (T2) and infected (T1) groups (p < 0.05). In addition, HDL levels in the T3 group (32.80 mg/100 g) remained higher than in the AGP-treated group (T2: 14.90 mg/100 g) but lower than in the negative control (T0: 61.48 mg/100 g). These results suggest that *L. paracasei* supplementation enhances the lipid profiles of broiler meat, reducing cholesterol and LDL while maintaining moderate HDL levels.

### Hematological profile

The hematological profiles of broilers differed between treatment groups (Table 3). Leukocyte, erythrocyte, hematocrit, lymphocyte, monocyte, and granulocyte counts showed no significant differences between groups (p > 0.05), suggesting that neither *L. paracasei* nor AGP treatment resulted in notable changes in these parameters. Significant differences (p < 0.05) were observed in hemoglobin and platelet (thrombocyte) levels. Hemoglobin levels were signif-icantly higher in the T0 group (negative control) (12.16 g/dL) than in the T3 group (probiotic) (9.84 g/dL; p < 0.05). Hematocrit levels showed a similar trend, with the T0 group having the highest values (32.06%), although the differences between treatments were not significant (p > 0.05). The T0 group had the highest platelet (thrombocyte) levels (283.00 × 10^3^/μL), which were significantly higher than those of the T2 (238.20 × 10^3^/μL) and T3 (224.80 × 10^3^/μL). The findings indicate that AGP (T2) and probiotic treatment (T3) had a modest impact on hematological parameters compared with AGP or untreated controls.

**Table 3:** Hematological profile.

Variable	T0	T1	T2	T3
Leukosytes (10^3^/μL)	28.84^a^ ± 1.70	23.82^a^ ± 3.45	21.56^a^ ± 0.49	21.04^a^ ± 1.27
Erythrocytes (10^6^/μL)	2.74^a^ ± 0.39	2.54^a^ ± 0.59	2.64^a^ ± 0.19	2.26^a^ ± 0.19
Hemoglobin (g/dL)	12.16^b^ ± 1.11	10.86^ab^ ± 2.47	11.64^ab^ ± 1.01	9.84^a^ ± 0.77
Hematocrit (%)	32.06^a^ ± 4.06	28.92^a^ ± 6.47	31.24^a^ ± 2.58	27.02^a^ ± 2.31
Thrombocytes (10^3^/μL)	283.00^c^ ± 8.77	257.40^b^ ± 20.98	238.20^a^ ± 1.48	224.80^a^ ± 9.93
Lymphocytes (%)	6.40^a^ ± 0.89	6.40^a^ ± 1.67	7.40^a^ ± 0.89	6.40^a^ ± 1.34
Monocytes (%)	7.40^a^ ± 0.54	7.40^a^ ± 1.34	6.60^a^ ± 1.14	7.00^a^ ± 2.00
Granulocytes (%)	86.20^a^ ± 1.09	86.20^a^ ± 1.09	86.00^a^ ± 1.41	87.20^a^ ± 0.83

^a,b,c^Different superscript letters within a column indicate statistically significant differences between treatment groups (p < 0.05)

### FCR, BWG, and FE

This study also evaluated the impact of *L. paracasei* supplementation on FCR, BWG, and FE in broilers challenged with *Escherichia coli*. Table 1 summarizes the results, indicating significant differences among the treatment groups for FCR, weight gain, and FE. The T3 group, which received 0.5% *L. paracasei* supplementation, demonstrated the most favorable FCR at 1.43, significantly surpassing the other groups (p < 0.05). Probiotic supplementation was observed to enhance feed conversion efficiency in broilers under infection conditions. The T0 group, representing the negative control (uninfected and untreated), exhibited a comparable FCR of 1.53, similar to that of T3, although without the advantages conferred by probiotic supplementation. The T1 group, infected with *E. coli* but without additive supplementation, exhibited an FCR of 1.57. Conversely, the T2 group, infected with *E. coli* and supplemented with AGP (zinc bacitracin), reco-rded the highest FCR at 1.60. However, no significant differences were detected among the T0, T1, and T2 groups (p > 0.05), suggesting that neither the absence of treatment nor AGP supplementation substantially improved FCR under infection conditions.

Broilers in the T3 group exhibited the highest BWG (520.20 g), with a statistically significant improvement over all other treatment groups (p < 0.05). These results indicate that *L. paracasei* supplementation markedly enhanced growth performance in *E. coli*-challenged broilers. Although the T0 group (uninfected, untreated) achieved a weight gain of 458.80 g, this was notably lower than the 520.20 g observed in the T3 group, emphasizing the probiotic’s efficacy. The T1 and T2 groups exhibited the lowest weight gains of 418.00 g and 432.40 g, respectively, with no significant differences between them. This finding suggests that probiotic supplementation in the T3 group was more effective in promoting weight gain than AGP supplementation.

Similarly, the T3 group achieved the highest FE (69.82%), significantly exceeding the other groups (p < 0.05). These findings demonstrate that *L. paracasei* supplementation improved feed utilization efficiency in broilers, particularly under *E. coli* infection. The FE of the T0 group (65.42%) was comparable to that of T3, implying that while the absence of infection supports better FE, probiotic supplementation further enhanced it. In contrast, both T1 and T2 groups showed reduced FE values of 63.91% and 62.70%, respectively, with no significant differences between them (p > 0.05). This indicates that *E. coli* infection negatively impacted FCR and FE. Probiotic supplementation (T3) effectively improved FCR and FE, whereas AGP supplementation (T2) did not lead to significant improvements in these parameters.

### SOD and MDA levels

The levels of SOD and MDA across treatment gro-ups are presented in Table 2. SOD, a critical antioxidant enzyme responsible for mitigating oxidative stress, exhibited significant variation among treatments. The T3 group exhibited the highest SOD activity (40.25 U/mL, p < 0.05), indicating that probiotic supplementation significantly enhanced the antioxidant defense system. In contrast, the T1 group (infected with *E. coli* without additives) exhibited the lowest SOD activity (6.41 U/mL). The T2 group (AGP supplementation) demonstrated higher SOD activity (14.78 U/mL) compared to T1, suggesting some improvement; however, SOD levels in T2 remained significantly lower than those observed in T3 (40.25 U/mL). The T0 group (negative control) exhibited a moderate SOD activity (19.61 U/mL), exceeding that of T1, potentially reflecting a natural antioxidant response in the absence of infection or supplementation.

MDA, a biomarker of lipid peroxidation and oxidative damage, also showed significant variation among groups. The T1 group exhibited the hig-hest MDA concentration (6.41 nmol/mL; p < 0.05), indicating increased oxidative stress under infection without intervention. In contrast, the T2 group (AGP supplementation) displayed significantly reduced MDA levels (2.27 nmol/mL), comparable to the T0 group (2.02 nmol/mL). The T3 group (probiotic supplementation) exhibited intermediate MDA levels (4.30 nmol/mL), suggesting partial protection against lipid peroxidation. The substantially lower MDA levels in the T2 group highlight the capacity of AGP to mitigate oxidative damage, resembling the natural oxidative balance observed in T0. However, comparable SOD activity between the T2 and T1 groups suggests that AGP alone provided limited enhancement of antioxidant defenses relative to probiotics.

### Cholesterol, HDL, and LDL levels in broilers

Cholesterol, HDL, and LDL levels in broiler meat are presented in Table 4. The T3 group (probiotic supplementation) recorded the lowest cholesterol levels (50.26 mg/100 g), significantly lower than those in all other groups (p < 0.05). These findings suggest that probiotic administration substantially reduced cholesterol concentrations in broiler meat. Conversely, the T2 group (AGP supplementation) exhibited the highest cholesterol levels (139.55 mg/100 g), followed by the T1 group (102.66 mg/100 g) and the T0 group (76.86 mg/100 g).

**Table 4:** Cholesterol, HDL, and LDL levels in broiler meat.

Variable	T0	T1	T2	T3
Cholesterol (mg/100 g)	76.86^ab^ ± 15.91	102.66^b^ ± 25.82	139.55^c^ ± 42.60	50.26^a^ ± 10.60
HDL (mg/100 g)	61.48^c^ ± 4.64	26.39^b^ ± 5.68	14.90^a^ ± 5.40	32.80^b^ ± 10.46
LDL (mg/100 g)	57.18^a^ ± 7.94	74.82^bc^ ± 0.93	82.94^c^ ± 18.78	66.00^ab^ ± 1.62

^a,b,c^Different superscript letters within a column indicate statistically significant differences between treatment groups (p < 0.05)

HDL concentrations were highest in the T0 group (61.48 mg/100 g), significantly exceeding HDL levels in all other groups. The T2 group had the lowest HDL concentration (14.90 mg/100 g), whereas the T1 (26.39 mg/100 g) and T3 (32.80 mg/100 g) groups displayed intermediate HDL values. LDL concentrations were highest in the T2 group (82.94 mg/100 g) and lowest in the T0 group (57.18 mg/100 g), with T1 (74.82 mg/100 g) and T3 (66.00 mg/100 g) groups showing intermediate LDL levels.

The results indicate that probiotic supplementation (T3) significantly reduced cholesterol (50.26 mg/100 g) and LDL (66.00 mg/100 g) compared with the AGP-treated (T2) and infected (T1) groups (p < 0.05). In addition, HDL levels in the T3 group (32.80 mg/100 g) were higher than those observed in the AGP group (T2: 14.90 mg/100 g) but lower than in the negative control (T0: 61.48 mg/100 g). These findings demonstrate that *L. paracasei* supplementation improves the lipid profiles of broiler meat, reducing cholesterol and LDL concentrations while maintaining moderate HDL levels.

### Hematological profile

The hematological profiles of broilers across treatment groups are summarized in Table 3. Leukocyte, erythrocyte, hematocrit, lymphocyte, monocyte, and granulocyte counts showed no significant differ-ences among groups (p > 0.05), indicating that neither *L. paracasei* supplementation nor AGP treatment significantly altered these hematological parameters.

However, significant differences (p < 0.05) were observed for hemoglobin and platelet (thrombocyte) levels. Hemoglobin concentrations were significantly higher in the T0 group (12.16 g/dL) compared to the T3 group (9.84 g/dL; p < 0.05). Hematocrit values showed a similar trend, with the T0 group exhibiting the highest values (32.06%), although differences across treatments were not statistically significant (p > 0.05). Platelet counts were also highest in the T0 group (283.00 × 10^3^/μL), significantly exceeding those observed in the T2 (238.20 × 10^3^/μL) and T3 (224.80 × 10^3^/μL) groups. These findings suggest that both AGP (T2) and probiotic (T3) treatments modestly influenced hemat-ological parameters, although no major hematological disturbances were detected compared to the control groups.

## DISCUSSION

### Broiler production and performance (FCR, BWG, and FE)

The improvements in growth performance observed in this study align with the findings presented in Table 4. As detailed in Table 1, *L. paracasei* supplementation (T3) resulted in significant enhancements in FCR, BWG, and FE among broilers challenged with *E. coli*. The T3 group exhibited the highest FCR (1.43) compared with the other treatment groups (p < 0.05), demonstrating that probiotics were more efficient than AGPs (T2) in improving feed conversion under infection conditions. Although the T0 group, which was uninfected and untreated, showed an FCR of 1.53 – comparable to T3 – this group lacked the additional benefits conferred by probiotic supplementation. The T1 and T2 groups, both infected with *E. coli*, exhibited elevated FCR values of 1.57 and 1.60, respectively, with T2 demonstrating the poorest performance. These results indicate that *L. paracasei* supplementation is more effective than AGP supplementation in optimizing feed conversion efficiency. This finding is consistent with earlier studies reporting that probiotics enhance feed conversion in broilers, whereas AGPs may increase feed intake without significantly improving nutrient absorption and metabolism [2, 23].

The T3 group exhibited the highest BWG (520.20 g), significantly exceeding all other groups (p < 0.05). These improvements in BWG and FE among probiotic-supplemented broilers suggest that *L. paracasei* enhances nutrient absorption and metabolism, possibly through modulation of the gut microbiota. Notably, probiotic supplementation yielded superior growth performance compared with AGP supplementation, reinforcing the potential of probiotics as alternatives to AGPs. While the T0 group demonstrated moderate growth performance in the absence of infection, the T3 group’s superior outcomes suggest that probiotics actively improve feed utilization even under infectious stress. No significant differences were observed between the T1 and T2 groups. These results imply that *L. paracasei* supplementation promotes feed intake and growth performance, particularly under stress conditions, a finding supported by Lin [24] and Hashemitabar and Hosseinian [25] demonstrating the efficacy of probiotics like *L. paracasei* in enhancing broiler growth under stressful situations such as infection. Thus, *L. paracasei* supplementation appears to be more effective than AGP supplementation in improving FCR and FE. Furthermore, probiotics represent a sustainable alternative to AGPs, enhancing poultry productivity while mitigating antibiotic resistance risks, and thereby positioning themselves as a promising strategy for modern poultry production.

### SOD and MDA levels

The effects of different treatments on oxidative stress, as presented in Table 2, revealed substantial changes in SOD and MDA levels among the groups. The T3 group exhibited the highest SOD activity (40.25 U/mL), indicating an enhanced antioxidant defense following *L. paracasei* supplementation. Previous studies have linked probiotic administration with increased antio-xidant enzyme activity, consistent with the SOD elevation observed in this study. For instance, supplementation with *L. paracasei* XLK401 was reported to significantly enhance hepatic SOD activity and reduce oxidative stress in hens [25]. In contrast, lower SOD levels were noted in the T1 (infection without additives) and T2 (AGP supplementation) groups, suggesting that AGPs did not stimulate antioxidant defenses as effectively as probiotics. Moderate SOD levels in the T0 group further suggest a baseline antioxidant response in the absence of interventions.

Significant variations in MDA levels, a marker of lipid peroxidation and oxidative damage, were also detected. The T1 group exhibited the highest MDA concentrations, indicating aggravated oxidative damage following *E. coli* infection without treatment. However, MDA levels were considerably lower in the T2 group supplemented with AGP, comparable to those in the T0 group. These results support earlier findings that probiotics can reduce oxidative damage and suggest that *L. paracasei* supplementation partially restores oxidative balance, similar to natural conditions [25]. The T3 group exhibited intermediate MDA levels (4.30 nmol/mL), suggesting partial protection against lipid peroxidation. These findings are consistent with prior studies demonstrating that *Lactobacillus* strains, including *L. paracasei*, play a pivotal role in mitigating oxidative stress, as evidenced by increased SOD act-ivity and reduced MDA concentrations. Furthermore, studies have shown that probiotics improve growth performance and oxidative status in broilers under prolonged heat stress [24], and supplementation with *L. salivarius* similarly enhanced antioxidant responses and growth performance in hens challenged with *E. coli* [26]. Collectively, these findings highlight the critical role of probiotics in reducing oxidative damage and enhancing poultry health.

### Cholesterol, HDL, and LDL levels in broilers

Lipid metabolism critically influences poultry health, meat quality, and subsequent consumer health outcomes. Table 4 presents the effects of different treatments on cholesterol, HDL, and LDL levels, revealing significant differences among the groups (p < 0.05). In this study, the T3 group (*L. paracasei* supplementation) exhibited the lowest cholesterol levels in broiler meat, corroborating previous research by Wang *et al*. [27] highlighting its cholesterol-lowering effects. In contrast, the T2 group (AGP supplementation) displayed the highest cholesterol concentrations, suggesting that AGPs alone are less effective in modulating lipid profiles compared to probiotic supplementation. Moreover, the T1 group (infection without treatment) exhibited the highest cholesterol and LDL levels, indicating that *E. coli* infection in the absence of intervention may exacerbate lipid dysregulation.

The T0 group (negative control) exhibited the highest HDL-C concentrations, suggesting that natural, untreated conditions favor better HDL-C regulation. In comparison, the T3 group showed reduced cholesterol and LDL levels while maintaining moderate HDL levels, reinforcing the potential of *L. paracasei* to improve lipid metabolism and mitigate risks associated with hypercholesterolemia. This is particularly important considering that probiotics enhance bile salt deconjugation, promoting increased cholesterol excretion, and lowering serum cholesterol concentrations [28]. In addition, evidence suggests that probiotic supplementation improves the LDL-to-HDL ratio, contributing to a reduced risk of cardiovascular diseases [29]. These findings collectively imply that probiotics, particularly when used alongside AGPs, offer a sustainable and effective strategy for improving lipid metabolism, poultry health, and the nutritional quality of poultry products.

### Hematological profile

Hematological parameters serve as important indicators of immune status and general health in broilers. Variations in these metrics can provide insights into physiological responses to dietary interventions or pathogenic challenges. In the current study (Table 3), no significant alterations (p > 0.05) were detected in leukocyte, erythrocyte, hematocrit, lymphocyte, monocyte, or granulocyte counts across the treat-ment groups, indicating that neither *L. paracasei* supp-lementation nor AGP treatment significantly affec-ted these hematological indices. These results align with previous study by Chaiyasut *et al*. [30] reporting that *L. paracasei* supplementation does not markedly alter the hematological profiles of broilers.

However, significant differences (p < 0.05) were observed in hemoglobin and platelet (thrombocyte) levels. The T0 group exhibited the highest hemoglobin and platelet values, suggesting better oxygen-carrying capacity and coagulation potential under unin-fected, untreated conditions. The T3 group (probiotic supplementation) exhibited the lowest platelet counts, indicating a mild influence of *L. paracasei* on thrombocyte production [31]. Nevertheless, the overall stability of erythrocyte and leukocyte counts, despite minor variations, suggests that neither probiotics nor AGPs induced substantial hematological disturbances. These findings corroborate previous observations that probiotic administration, including that of *L. paracasei*, has negligible impacts on the hematological parameters of broilers.

## CONCLUSION

The findings of this study demonstrate that *L. paracasei* supplementation significantly improved growth performance, antioxidant capacity, and lipid metabolism in broilers challenged with *Escherichia coli*. Specifically, probiotic treatment led to a markedly lower FCR (1.43), enhanced BWG (520.20 g), and improved FE (69.82%) compared with both AGP-treated and untreated infected groups. Antioxidant defense mechanisms were strengthened, as evidenced by higher SOD activity and reduced MDA levels in probiotic-supplemented birds. Furthermore, *L. paracasei* supplementation resulted in lower total cholesterol and LDL levels, while mainta-ining moderate HDL concentrations, thus improving the overall lipid profile of broiler meat. Hematological assessments revealed that probiotic administration did not adversely affect immune-related parameters, ensuring its safety for poultry application.

The practical implications of these findings are significant, indicating that *L. paracasei* supplementation could serve as a sustainable alternative to AGPs in poultry production. Its ability to enhance growth, oxidative balance, and meat quality under infection pressure supports the transition toward antibiotic-free broiler farming, aligning with public health goals and regulatory restrictions on AGP usage.

The primary strength of this study lies in its comprehensive assessment of production performance, oxidative status, lipid metabolism, and hematological profiles under controlled experimental conditions mimicking field-relevant *E. coli* infections. The integration of growth, biochemical, and health parameters provides robust evidence supporting the multifunctional benefits of probiotic supplementation.

Nevertheless, the study presents certain limita-tions. It was conducted under experimental conditions with a specific pathogen challenge, and thus, field validation under diverse farming environments is warr-anted. In addition, the effects of varying probiotic dosages, different probiotic strains, and longer supplementation periods were not assessed and may influence the observed outcomes.

Future research should focus on evaluating the synergistic effects of multi-strain probiotics, exploring optimal dosing strategies, and assessing long-term impacts on poultry gut microbiota composition and resistance to multiple pathogens. Large-scale field trials across diverse production systems are also needed to confirm the practical applicability and economic feasibility of *L. paracasei* supplementation in commercial broiler production.

## AUTHORS’ CONTRIBUTIONS

WPL and LM: Collected and analyzed the data. ALS and ER: Drafted the manuscript. ABY and ZNAR: Analyzed and interpreted the data. MAF and HIS: Conceptualized and designed the study. EFL, ARK, and MS: Revised the manuscript. TDM, ZAB, ALS, and ER: Analyzed the data and revised the manuscript. All authors have read and approved the final manuscript.
